# Gender non-binary adolescents’ somatic and mental health throughout 2020

**DOI:** 10.3389/fpsyg.2022.993568

**Published:** 2022-12-23

**Authors:** Catrin Johansson, Carina Kullgren, Kourosh Bador, Nóra Kerekes

**Affiliations:** ^1^Department of Health Sciences, University West, Trollhättan, Sweden; ^2^Agera KBT AB, Gothenburg, Sweden; ^3^Center for Holistic Psychiatry Research (CHoPy), Mölndal, Sweden

**Keywords:** affect, adolescents, mental health, non-binary gender, personality, risk behaviors, somatic health, victimization

## Abstract

**Background:**

Non-binary gender adolescents are particularly vulnerable and more likely to be exposed to several socio-psychological difficulties and disorders. It is vital to discover and act on the vulnerabilities they encounter. The present study aims to describe the somatic and mental health, affect state, frequency of risk behaviors, victimization and negative psychosocial factors, as well as the personality profiles of non-binary adolescents. In this study the concept of gender non-binary is used and captured respondents who selected “neither of these” as their gender from the possible options (female/male/neither of these).

**Materials and methods:**

Data was collected between September 2020 and February 2021 in Sweden, Morocco, Serbia, Vietnam, and the United States. The cross-sectional, retrospective study utilized the electronic version of the Mental and Somatic Health without borders (MeSHe) survey. From the over 5,000 responses of 15–19-year-old adolescents, 58 respondents identified as being non-binary, and built our study population. Their data was analyzed with descriptive statistic methods.

**Results:**

Close to a fourth of adolescents identifying as non-binary reported the existence of at least one somatic disease. The most prevalent somatic disease was allergies. Almost one-third had suffered from pain either often or all the time in the past 12 months. The highest levels of perceived psychological distress were measured using obsessive–compulsive symptoms, depression, and interpersonal sensitivity. The average level of alcohol and drug use during the past 12 months was low. About 40% of non-binary adolescents reported having experienced physical abuse, and half of them experienced psychological abuse at some point in their lives. Seventeen percent reported living with adults with alcohol-use problems. Non-binary adolescents’ personalities were found to be dominated by high scores in Openness, Neuroticism, and Agreeableness.

**Conclusion:**

This study presents a detailed biopsychosocial picture of a multinational sample of non-binary adolescents. Our study suggests that awareness and support are required from all fields of society, including family, school, healthcare, and educational institutions, for cis-normative culture to progress toward a greater understanding of and respect for gender diversity.

## 1. Introduction

People who identify as gender non-binary face significantly different life situations and well-being states compared to those who identify as gender binary. Current research agrees that non-binary gender individuals, especially adolescents and young adults, are particularly vulnerable and more likely to be exposed to several socio-psychological difficulties and disorders (Aparicio-García et al., 2018; de Graaf et al., 2021). Although non-binary gender identities are not new, general awareness of the group and its vulnerabilities has recently increased (Vijlbrief et al., 2020). During the last few decades, gender terminology and identities have diversified and become more visible in the public space (Richards et al., 2016). Consequently, the non-binary gender has become more widely accepted, although this varies across the world. The actual distinction and categorization of the group has a brief history in the public mind, and the same goes for the body of accumulated knowledge on the topic, although research has increased significantly in recent years. Healthcare professionals, especially in psychiatry, have the challenge and responsibility to deepen their understanding and knowledge about non-binary gender adolescents to be able to provide them with appropriate care.

Being familiar with diverse, evolving terminology and its meaning is a prerequisite for improved understanding. The non-binary gender is defined as a form of gender incongruence, which nowadays refers to the difference between the gender assigned at birth and perceived gender identity and expression (Andersen et al., 2020). The term is used as an umbrella concept and covers a spectrum of diverse gender identities, including bi-gender, gender fluid, a-gender, genderqueer, and others (Richards et al., 2016; Liszewski et al., 2018; Paz-Otero et al., 2021). As Richards et al. (2016) note, what unites them is the fact that they are distinct from the gender binary. The inability or unwillingness to identify as either a woman or a man, preferring to experience oneself as someone between the categories, as both, or as someone beyond the conventional gender matrix, means that they transgress and thus challenge established and normalized gender expectations of heteronormativity (Vijlbrief et al., 2020).

In the present study, the concept of gender non-binary was considered the most appropriate to describe those who chose the last option when asked about gender in the survey. The three options provided were “female,” “male,” and “neither of these.” We do not know how the adolescents read the question, but regardless of whether they interpreted it as being biological or as social gender, they chose to stand outside of the established gender dichotomy.

Deviating from prevailing gender norms may have a negative impact on non-binary people’s personal development and well-being, especially concerning their mental health, social relations, personal networks, and work possibilities. Besides worsened well-being, they have an increased risk of exposure to harassment and violence (Aparicio-García et al., 2018; Newcomb et al., 2020).

It is essential to discover and act on the vulnerabilities faced by individuals who identify as gender non-binary, especially during adolescence. Many of them must deal with more psychosocial problems than gender binary/cisgender youth, ranging from inequality, prejudice, social stigma, and discrimination to low self-esteem, mental health complaints, and suicide (Richards et al., 2016; Andersen et al., 2020). Although studies differ in their methodological allocations, samples, and reported figures, they largely show similar problematic results regarding non-binary people’s social situations and health. For example, compared to gender binary/cisgender people, the levels of depression faced by gender non-binary individuals are disproportionately high (Newcomb et al., 2020; Moore et al., 2021). Recent studies indicate that non-binary adolescents also have an increased chance of being higher on the autism spectrum, having eating disorders, and facing weight manipulations, especially those who are assigned female at birth (Paz-Otero et al., 2021).

One aspect of complexity that characterizes the group is that non-binary people must constantly deal with contradictory processes of inclusion and exclusion (Vijlbrief et al., 2020). The elevated level of victimization of this group means that they are forced to constantly take social risks. For example, when it is impossible to predict how and in what contexts the environment will react to displayed gender identity, a constant readiness to be treated as deviant, as “the other,” is forced on them. Gender non-binary youth can never take for granted that they will pass as “normal” and “understandable” in new or unknown contexts. They must constantly expose themselves to the risk of being rejected, excluded, or stigmatized. Living with such constant insecurity can be socially and mentally stressful and increase their level of perceived psychological distress. In addition, an increased prevalence of risk behavior among non-binary adolescents compared to gender binary/cisgender ones has been identified, such as increased drug use and sexual behaviors (Eisenberg et al., 2017). As part of the “coming out” process and attempting to find ways to understand and manage their own life situation, gender non-binary adolescents require extra social and psychological support.

Several studies also show that the types of problems affecting non-binary people have worsened, often significantly, during the COVID-19 pandemic (Mirabella et al., 2021; Perl et al., 2021). In addition, the group’s social support decreased during the pandemic, which can be attributed to the fact that non-binary people are more dependent on self-chosen families compared to other gender minorities. This can result in social isolation, leading to 29% increased mortality (Holt-Lunstad et al., 2015; López-Sáez and Platero, 2022).

To be able to complete previous research the present study aims to describe the somatic and mental health, affect state, frequency of risk behaviors, victimization and negative psychosocial factors, as well as the personality profiles of adolescents who identify as gender non-binary in a multinational study population. Our data carries information about how these adolescents felt, behaved and what type of experiences they had during the year 2020, which was the first year of the COVID-19 pandemic. Therefore, we show the COVID-19 pandemic’s influence reported by adolescents on these variables. We also aim, to set the study’s descriptive results in the context of knowledge from a dominantly gender binary-normative academic research and shed light on future challenges in the discussion. Our intention is to contribute to the research and knowledge in the field, especially for healthcare professionals, social workers, and teachers who interact with this vulnerable group of young people.

## 2. Materials and methods

### 2.1. Study design and procedure

This study has a cross-sectional design and is part of a large international project: the Mental and Somatic Health without borders (MeSHe) project.^1^ The countries that are part of the MeSHe project are Sweden, Serbia, Morocco, Vietnam, and the United States. For data collection, self-report instruments on an electronic survey (eMeSHe) were used. They were available in five languages. The participants were secondary high school students, adolescents between the ages of 15 and 19, who were selected by convenient sampling using a non-probabilistic method. First (Fall 2020), we reached out in designated cities of participating countries to high school teachers and heads of schools to contact high school students. However, because of the different restrictions and approaches during the COVID-19 pandemic from some of the participating countries the response rate was very low. In these countries (Sweden, United States, and part of the data in Morocco) students were reached *via* Facebook and Instagram (Winter 2020).

The online survey consists of validated instruments, where adolescents rated their own physical and mental health, their aggressive, antisocial, and self-harm behaviors, their personality, the intensity and frequency of leisure-time physical activity, and their general affect state considering the past 12 months (retrospective). They also answered questions about the extent to which the COVID-19 pandemic changed their behavior, mood, psychosocial functioning, and victimization. The completion of the survey is estimated to take 35–45 min. The eMeSHe survey was open to answers between September 2020 and February 2021.

Participation was voluntary, and the survey was answered anonymously. Ethical permission to conduct the study has been given by each country’s respective ethics board, ensuring that the study follows each country’s law about not including minors (up to 15 years old) in the study. If the online survey gave rise to any questions or concerns of the participants, the end of the survey contained information and links to relevant support organizations in each participating country.

### 2.2. Study population

The study population is described in detail in Kerekes et al. (2021b). Briefly, respondents were recruited *via* their schools (in Vietnam and Serbia, part of the data in Morocco, and a minor part of the data in Sweden and the United States) and by social media (Sweden, the United States, and part of the data in Morocco). The national samples were not representative of the nation (see a more detailed description in Kerekes et al., 2021b).

During the study period (September 2020–February 2021), 5,341 complete responses were received on the eMeSHe survey from the five participating countries.

After exclusion of those under or over the approved age-span of the study, the final number of responses was 5,114 (1,534 from Vietnam, 1,108 from Serbia, 1,608 from Sweden, 541 from Morocco, and 323 from the United States), of which 37.0% were male, 61.8% were female, and 1.2% selected “neither of these” gender category. The mean age of the respondents was 16.69 (SD = 1.01).

For the present study, the data of those (*N* = 58) who selected “neither of these” as their gender from the possible options (female/male/neither of these), from here on referred to as gender non-binary adolescents, was utilized. We cannot be sure which gender category (female/male/neither of these) transgender adolescents chose, leaving the possibility that they are included in our non-binary category. We know that there were five adolescents from the 5,114 respondents, indicating in their psychiatric diagnosis transsexuality, and they have identified with male gender. The present study population (gender non-binary adolescents, *N* = 58) had a mean age of 16.48 years (Md = 16, SD = 0.99). The country of residence was distributed as follows, Sweden (*n* = 15, 26%), Morocco (*n* = 4, 7%), Serbia (*n* = 7, 12%), Vietnam (*n* = 15, 26%), and the United States (*n* = 17, 29%).

### 2.3. Measures

#### 2.3.1. Background questions

The eMeSHe survey starts with some background questions, which include information about gender, age, country of residence, the existence of medically diagnosed psychiatric disorders, physical disability, the existence of negative psychosocial factors (living with an adult with an alcohol use problem, living with an adult with a drug use problem, any experience of psychical abuse, or any experience of psychological abuse), and information about the frequency and intensity of generally experienced pain over the past year.

#### 2.3.2. MeSHe health survey

The MeSHe health survey consists of questions through which adolescents report the existence of defined somatic diseases and complaints. It has been previously used in adolescent populations (Zouini et al., 2019a; Kerekes et al., 2021a), and its test–retest variability is proven to be acceptable (Zouini et al., 2019a).

#### 2.3.3. The Godin–Shephard leisure-time physical activity questionnaire

In the Leisure-Time Exercise Questionnaire respondents indicate the frequency of “strenuous,” “moderate,” and “light,” physical activities/exercises performed weekly. A total score is calculated by the following rule: strenuous/exhausting (9 METs × times/week) + moderate (5 METs × times/week) + light (3 METs × times/week; Godin and Shephard, 1985).

#### 2.3.4. Brief symptom inventory

The Brief Symptom Inventory (BSI) is a measure of self-perceived mental or psychological distress (Derogatis and Melisaratos, 1983). It consists of 53 items divided into nine domains (primary symptoms scales). The sum of the responses builds up the General Severity Index (GSI). Each item measures the extent to which the adolescent experiences the given statement. The reliability of the BSI instrument was recently tested in a multinational sample, where it showed acceptable reliability for each primary domain, except that of Hostility (Nguyen et al., 2022). Therefore, in the present study, we report scores on eight of the primary domains (we do not report scores on Hostility). We report the GSI scores without the scores on Hostility as well.

#### 2.3.5. Alcohol use disorder identification test

The World Health Organization (WHO) developed the Alcohol Use Disorder Identification Test (AUDIT) in 1993 (Saunders et al., 1993). It identifies early hazardous and harmful drinking. The instrument contains 10 items rated on a 4-point Likert scale, with a total score ranging from 0 to 40. There are three items that focus on alcohol use, four on dependence, and three about problems related to the alcohol habit. Alcohol problems were previously assessed among the general population (Aalto et al., 2009), the elderly (Aalto et al., 2011), and adolescents (Rumpf et al., 2013; Hasler et al., 2014). On the AUDIT scale there are previously validated gender (male/female) specific cut-offs to identify those with hazardous, harmful, or dependent drinking (Saunders et al., 1993). However, according to our knowledge no cut-off validated to other than male and female genders, and new studies point out that a range of AUDIT cut-off scores appear to be suitable based on cultural contexts, therefore we will not attempt to describe our study populations in this aspect, and we warn the readers to do that. Cronbach’s alpha in the present study for AUDIT was 0.78.

#### 2.3.6. Drug use disorder identification test

The Drug Use Disorder Identification Test (DUDIT) contains 11 questions that capture the frequency of drug use during the last year using self-reported data. Items from 1 to 9 are rated on a 5-point Likert scale, while items 10 and 11 are rated on a 3-point Likert scale. The DUDIT total score is calculated using the sum of all items; scores range between 0 and 44. DUDIT has been previously used in the adolescent population (Hillege et al., 2010; Hasler et al., 2014). On the DUDIT scale there are previously validated gender (male/female) and even culture specific cut-offs to identify those with drug use dependence in the different countries. However, no cut-off validated to other than male and female genders, and according to our knowledge no cross-cultural cut-off exist to us in our multinational sample. Therefore, we will not attempt to describe our study populations in this aspect, and we warn the readers to do that. Cronbach’s alpha in the present study was 0.94.

#### 2.3.7. Adolescent-adapted life history of aggression

The Adolescent-Adapted Life History of Aggression (AA-LHA) is a version of Coccaro’s Life History of Aggression self-reported scale (Coccaro et al., 1997). It is adapted for a multinational, adolescent population (Stevanovic et al., 2022). It measures the occurrence of aggressive and antisocial behaviors from the age of 13. In a recent study, we analyzed cross-cultural validity and the optimal structure of the LHA, which resulted in the AA-LHA (Stevanovic et al., 2022). The AA-LHA contains nine questions that can be divided into two subscales, the Aggression subscale with five questions, and the Antisocial behaviors/Consequences subscale with four items.

#### 2.3.8. COVID-19-related questions

The eMeSHe survey was completed with COVID-19-related questions at the end of 2020. These include items about changes caused by the COVID-19 pandemic in adolescents’ risk behaviors, general well-being, and victimization. It also includes questions about the frequency of victimization in the respondents’ whole lives. Detailed descriptions of the questions can be read at Kerekes et al. (2021b). In the present study, we use, from these questions, the description of risk behaviors (alcohol and drug use), norm-breaking behaviors (as a complement to the information gathered using other instruments, such as AUDIT, DUDIT, and AA-LHA), and the frequency of victimization during the COVID-19 pandemic and in the overall perspective of life.

#### 2.3.9. Positive and negative affect schedule—Expanded form 30 items

The Positive and Negative Affect Schedule-Expanded form 30 items (PANAS-X30) (Watson et al., 1988) is an instrument that allows participants to rate the degree to which they experienced the listed 30 feelings over the past year. The positive affect scale consists of 15 adjectives, 10 of which describe high-arousal experiences and five of which describe low-arousal experiences, such as feelings of energy, strength, inspiration or calmness and serenity. The negative affect scale, also consisting of 15 adjectives, 10 with high-arousal experiences and five with low-arousal experiences, describes undesirable inner experiences such as anger, irritability, guilt, shame, or nervousness.

#### 2.3.10. Big five inventory

The Big Five Inventory (BFI) comprises of 44 short-phrase items that measure the degree of five scales. These are Extraversion (E; 8 items), Agreeableness (A; 9 items), Conscientiousness (C; 9 items), Neuroticism (N; 8 items), and Openness (O; 10 items). Participants were asked to express their degree of agreement or disagreement with each item presented on a 5-point Likert scale, ranging from disagree strongly to agree strongly. Examples of items from each of the scales are “Is talkative” (Extraversion), “Tends to find fault with others” (Agreeableness), “Does a thorough job” (Conscientiousness), “Is depressed, blue” (Neuroticism), and “Is original, comes up with new ideas” (Openness) (John and Srivastava, 1999).

### 2.4. Analyses

Descriptive statistics with minimum and maximum scores, mean (M), median (Md), standard deviation (SD), standard error (SE) and frequencies (%) with 95% confidence interval (CI) were used at both the scale and item levels. The Chi^2^ test was used to determine the level of significance in the comparison of negative and positive affect levels within the non-binary adolescent group. The significance level was set at *p* < 0.05. All analyses were performed using SPSS version 28.

## 3. Results

### 3.1. Prevalence of somatic complaints and pain

In the multinational sample of adolescents identifying as gender non-binary, the most prevalent somatic complaints were allergies (31%), asthma (21%), and migraines (9%) (Table 1). Interestingly, the proportion of adolescents who indicated that they do not know if they have a specific disease or not was the highest in these three diseases and skin and celiac diseases (Table 1).

**Table 1:** Prevalence of somatic diseases in the multinational sample of gender non-binary adolescents (*N* = 58).

	*n*	Yes (*n*)	95% CI	I do not know % (*n*)	95% CI
Cancer	58	0.0% (0)	0.0–6.2%	1.7% (1)	0.0–9.2%
Epilepsy	57	3.5% (2)	0.4–12.1%	0.0% (0)	0.0–6.3%
Migraine	57	8.8% (5)	2.9–19.3%	10.5% (6)	0.4–21.5%
Diabetes	57	1.8% (1)	0.0–9.4%	1.8% (1)	0.0–9.4%
Celiac disease	58	1.7% (1)	0.0–9.2%	6.9% (4)	1.9–16.7%
Thyroid disease	54	1.9% (1)	0.0–9.9%	1.9% (1)	0.0–9.9%
Rheumatological disease	58	0.0% (0)	0.0–6.2%	1.7% (1)	0.0–9.2%
Skin diseases	57	7.0% (4)	1.9–17.0%	10.5% (6)	4.0–21.5%
Tuberculous	57	0.0% (0)	0.0–6.3%	0.0% (0)	0.0–6.3%
Asthma	58	20.7% (12)	11.2–33.4%	6.9% (4)	1.9–16.7%
Allergies	58	31.0% (18)	19.5–44.5%	12.1% (7)	5.0–23.3%

CI, Confidence interval.

There were 38 adolescents (53.5% of the study population) who responded to each of the questions about somatic diseases with a yes or no. More than half of these (52.6%, *n* = 20) did not indicate any somatic complaints, 23.7% (*n* = 9) reported having one disease or complaint, 18.4% (*n* = 7) reported having two, 2.6% (*n* = 1) had three, and 2.6% (*n* = 1) had four coexisting somatic diseases.

Problems with diarrhea and constipation for a period longer than 14 days had a prevalence of 7% (CI = 2.2–19.2%) and 16% (CI = 8.6–31.4%), respectively, and in each category an additional 13.8% (*n* = 8, CI = 2.6–2.54%) indicated that they do not know.

Almost one third (28%, CI = 17–41%) of adolescents reported suffering from pain often or all the time, 53% (CI = 39–66%) reported pain sometimes and rarely, while only 19% (CI = 10–32%) indicated that they never experienced pain over the past 12 months. The average level of pain intensity in the sample was 3.98 (SD = 2.36, SE = 0.33, Md = 4.0). The different types of pain reported were, for example, headache, lower back pain, joint pain, and stomach pain.

Twelve percent (*n* = 7, CI = 5–23%) of the adolescents reported that they had a physical disability that prevented them from being physically active. The mean value of weekly leisure time activity was 33.1 (SD = 33.6, SE = 4.61, Md = 22, range from 0–136).

### 3.2. Prevalence of psychiatric disorders

**Figure 1:**
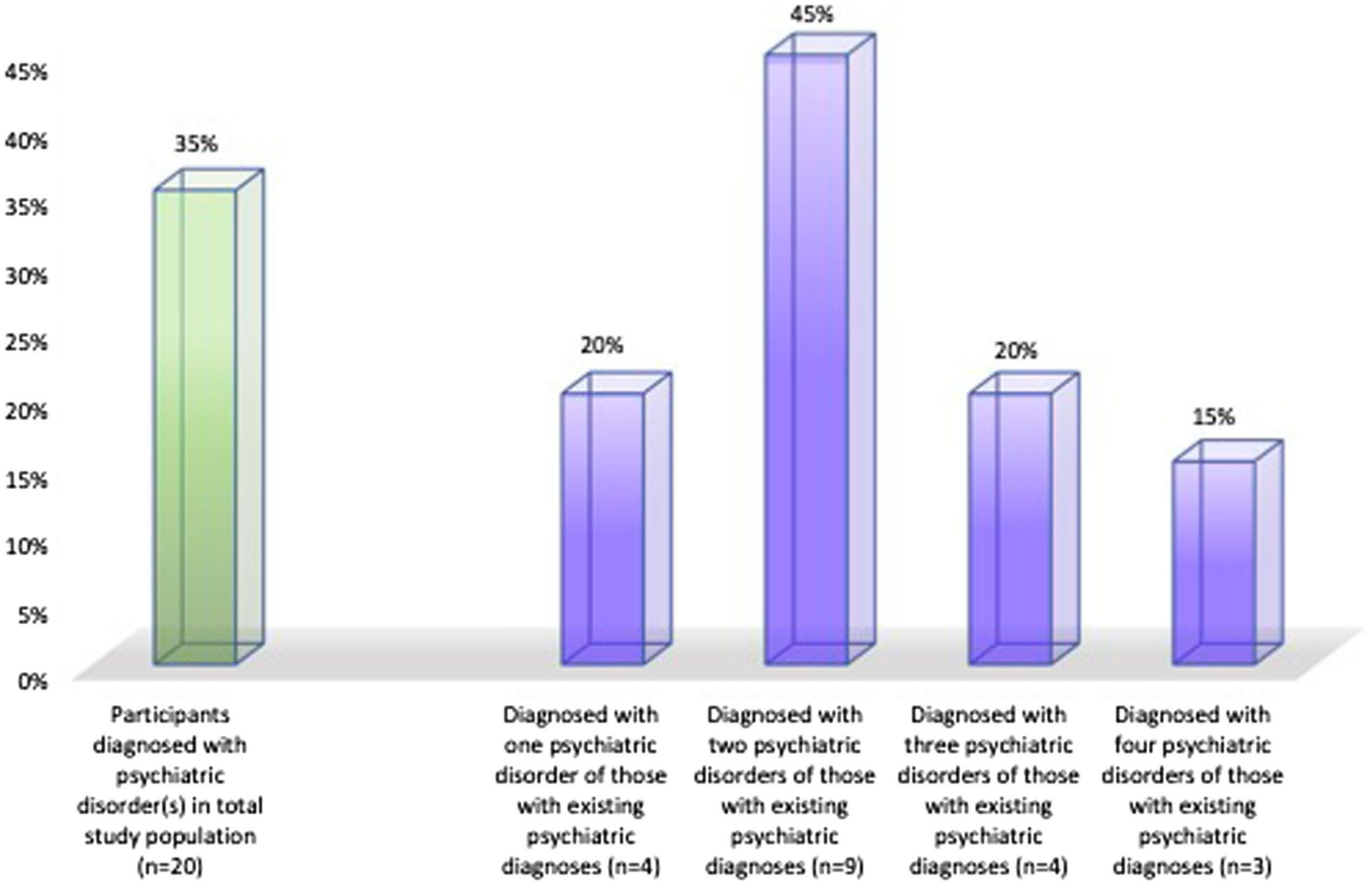
The prevalence of those with at least one psychiatric disorder (green column) in the whole study population (*n* = 55), and the prevalence of one, two, three and four psychiatric diagnose (purple columns) within those with any psychiatric diagnosis (*n* = 20).

Over half (65%, *n* = 35, CI =46–73%) of the adolescents had never been diagnosed with a psychiatric disorder. The remaining 35% (*n* = 20, CI = 27–54%) had been diagnosed with psychiatric disorder. These were most often anxiety disorders (80%, *n* = 16), depression (75%, *n* = 15), neurodevelopmental disorders such attention deficit hyperactivity disorder (ADHD) and/or autism spectrum disorder (ASD; 35%, *n* = 7), obsessive compulsive disorder (OCD; 20%, *n* = 4), post-traumatic stress disorder (10%, *n* = 2), and one adolescent each indicating self-harm and bipolar disorders. Of these adolescents, 80% (*n* = 16) had more than one coexisting psychiatric diagnosis (Figure 1).

The prevalence of adolescents currently taking prescribed (any, including those prescribed for somatic diseases) medication was 26% (CI = 16–40%).

### 3.3. The level of perceived psychological distress

The overall psychological distress level (GSI) was 1.60 (SD = 0.95, SE = 0.14, min–max: 0–4) in the study population. The highest level of distress was measured in the obsessive–compulsive symptoms of BSI (symptoms such as trouble remembering things, feeling blocked in getting things done, having to check and double check what you do, difficulty in making decisions, your mind going blank, trouble concentrating) and depression followed by interpersonal sensitivity domains. The lowest score was reached in the somatization domain (Table 2).

**Table 2:** The level of psychological distress among non-binary adolescents (*N* = 58).

Domain	*n*	*M* (SD/SE)
Somatization	54	1.30 (1.08/0.15)
Obsessive compulsive	54	2.11 (1.22/0.17)
Psychoticism	55	1.71 (1.13/0.15)
Depression	55	2.11 (1.17/0.16)
Interpersonal Sensitivity	55	1.94 (1.26/0.17)
Phobic anxiety	56	1.51 (1.14/0.15)
Anxiety	56	1.78 (1.17/0.16)
Paranoid Ideation	57	1.60 (1.22/0.16)
GSI (without Hostility)	46	1.60 (0.95/0.14)

*M*, Mean; SD, standard deviation; SE, standard error.

### 3.4. Risk behaviors

Over 60% (62.1%; *n* = 36 on the COVID-related questions and 61.8%; *n* = 34 on AUDIT) of adolescents indicated that they have never used alcohol. Twelve adolescents (20.7%) indicated that their usage did not change after the outbreak of the COVID-19 pandemic. Three (5.1%) indicated that their alcohol consumption decreased while seven (12.1%) indicated that their alcohol use increased. The majority (87.9%; *n* = 51 on the COVID-related questions, and 92%; *n* = 52 on the DUDIT) indicated that they have not used drugs during the past 12 months or ever. Two (5.2%) adolescents’ illegal drug use did not change after the COVID-19 outbreak, while three (1.7%) reported greatly decreased use and while two (5.2%) reported greatly increased use.

The average level of the AUDIT score (*M* = 2.59, SD = 4.33, SE = 0.63, Md = 0) and DUDIT score (*M* = 1.08, SD = 3.46, SE = 0.49, Md = 0) was low (Table 3). The average frequency of aggressive behaviors since they turned 13 years of age (on the AA-LHA Aggression subscale) was 9.82 (SD = 6.49, SE = 0.87, Md = 9), and the frequency of antisocial behavior (on the AA-LHA Antisocial behaviors/Consequences subscale) was 1.25 (SD = 2.33, SE = 0.32, Md = 0; Table 3).

**Table 3:** Self-reported risk behaviors among non-binary adolescents (*N* = 58).

	*n*	*M* (SD/SE)	Md	Min–max
AA-LHA Aggression	55	9.82 (6.49/0.87)	9	0–25
AA-LHA Antisocial behavior/Consequences	57	1.25 (2.33/0.31)	0	0–9
AUDIT total	47	2.59 (4.33/0.63)	0	0–22
DUDIT total	50	1.08 (3.46/0.49)	0	0–21

AA-LHA, Adolescent Adjusted Life History of Aggression; AUDIT, Alcohol Use Disorder Identification Test; DUDIT, Drug Use Disorder Identification Test, *M*, Mean; Md, Median; SD, standard deviation; SE, standard error.

### 3.5. The presence of negative psychosocial factors and victimization

About 20% (*n* = 11) reported that someone had physically abused them at least once in the past 12 months. This included being hit, kicked, or subjected to other forms of violence that caused injuries without the need of hospitalization. 40% (*n* = 23) reported that they have experienced physical abuse (being pushed, kicked, beaten, slapped, etc.) in their life. A fourth (25%, *n* = 16) have been threatened and felt seriously afraid in the past 12 months, while half (50%, *n* = 29) indicated that they have experienced psychological abuse (have been threatened, forced to do something that felt wrong, been violated by humiliating and insulting words) at some point in their life. One fourth (25%, *n* = 14) of them reported that they had been grouped or touched in a sexual manner without their consent. A fourth (25%, *n* = 14) reported that someone had written offensive things about them online, and 16% (*n* = 9) reported that someone had uploaded pictures or videos about them without their consent on the internet.

Considering negative psychosocial factors in their micro-environment (family) 17% (*n* = 10, CI = 9–29%) reported living with adults with an alcohol use problem. Four of them (40%) also reported experiencing both physical and psychological abuse, and another 40% reported no experience of physical or psychological abuse. Two did not respond to the question about abuse. One adolescent reported living with an adult with a drug use problem. This adolescent also reported experiencing of physical and psychological abuse.

### 3.6. Prevalence of positive and negative affect

Adolescents reported a significantly higher level of negative than positive affect states (*p* = 0.05). The differences focused on low arousal emotions (*p* < 0.001) (Table 4).

**Table 4:** Positive, negative, activated and deactivated affect among non-binary adolescents.

	Negative		Positive		Value of *p*
	*n*	*M* (SD/SE)	*n*	*M* (SD/SE)	
High arousal	52	26.63 (10.15/1.41)	54	24.94 (8.64/1.18)	0.43
Low arousal	54	16.68 (5.17/0.70)	55	12.33 (5.72/0.77)	<0.001
Total	50	43.38 (14.92/2.11)	54	37.24 (13.50/1.84)	0.05

*M*, Mean; SD, standard deviation; SE, standard error.

### 3.7. Personality profiles

**Figure 2:**
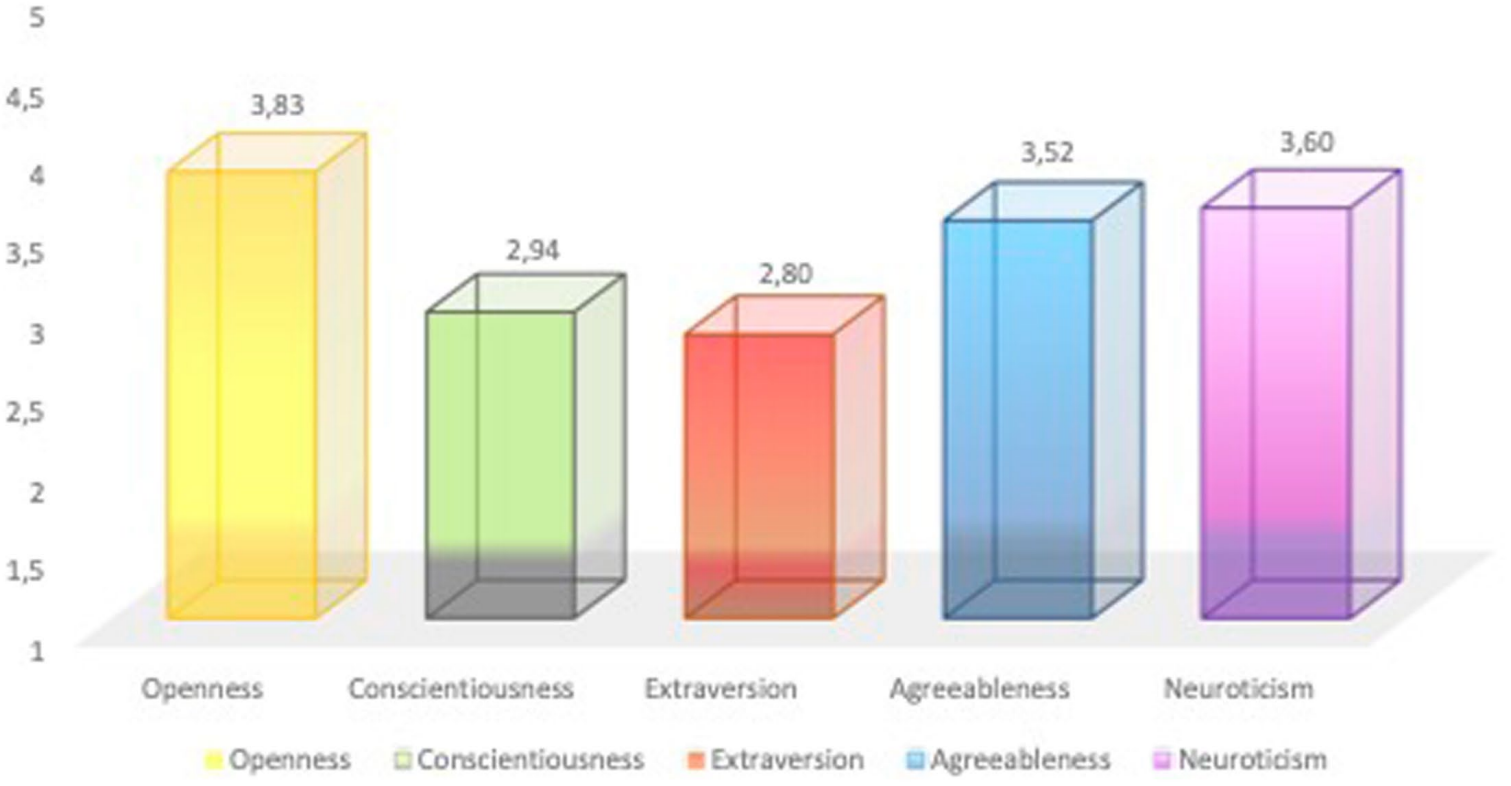
The levels of personality domains captured by the Big Five Inventory among gender non-binary adolescents (*N* = 52).

Non-binary adolescents had an average score on the Openness domain at 3.83 (SD = 0.72), the Conscientiousness domain at 2.94 (SD = 0.67), the Extraversion domain at 2.8 (SD = 0.84), the Agreeableness domain at 3.52 (SD = 0.55), and the Neuroticism domain at 3.6 (SD = 0.83). This was according to the Big Five Inventory (Figure 2).

## 4. Discussion

Most of the previous research studied the societal and mental health impacts being gender non-binary had on individuals (Richards et al., 2016; Eisenberg et al., 2017; Liszewski et al., 2018; Frost et al., 2019; Newcomb et al., 2020; Surace et al., 2021). According to our knowledge, we have yet to find a study that provides a more holistic picture of somatic and mental health, affect state, frequency of risk behaviors, the prevalence of negative psychosocial factors, and the level of victimization while capturing non-binary adolescents’ personality profiles. The present study offers all these without claiming a complete description of the biopsychosocial characteristics of these adolescents. To be able to contribute to the field of knowledge at this stage, in the following discussion we problematize the findings in relation to previous research mainly based on gender binary data.

### 4.1. Somatic health and perceived pain

The present study’s results showed that more than half of the adolescents did not indicate any somatic diseases, while about 25% indicated only one. The rest reporting having two or more coexisting somatic diseases. One third (31, 95% CI: 19.5–44.5%) of the adolescents reported having existing allergies and about one fifth (21, 95% CI: 11.2–33.4%) reported having asthma. In the interpretation of these results, we must point out that the confidence intervals were wide in our study, based on the considerable small study population, therefore future studies with higher sample size may find that the prevalence of these somatic diseases are closer to those found in binary adolescent population, but it may deviate even more drastically. A new study described the prevalence of asthma and allergies in a multinational sample of 15–19-year-old adolescents with over 4,800 male and female respondents (unpublished data from the MeSHe project). In that study, 24% of adolescents report having allergies and 10% report having asthma. These results indicate that possibly a higher proportion of gender non-binary adolescents suffer from these inflammatory conditions. Asthma and allergies, including food allergies, have increased over the past half decennium and became the most prevalent childhood chronic illnesses in developed countries (Locksley, 2010). While there is an element of genetic susceptibility behind these diseases, nobody questions the strong impact of the environment resulting in such increasing prevalence. Perceived stress due to several mechanisms can cause an imbalance in the immune system and lead to allergic reactions (Dave et al., 2011) or the exacerbation of asthma (Chen and Miller, 2007). It is also seen that allergies increase the odds of depression, anxiety, and personality and stress disorders in adult populations (Kelly et al., 2019). This is in concordance with our findings (discussed in detail in the next sections) that these psychiatric disorders are the most commonly reported in our study population.

When asking adolescents about how often they experienced pain during the past 12 months, 28% reported suffering from pain often or all the time. It most often consisted of headaches, lower back pain, joint pain, and stomach pain. This is comparable with a Norwegian study of 14–15-year-old adolescents that found that 29% suffered from prevalent, persistent pain (Mikkelsen et al., 2021). The median intensity of the pain in the Norwegian study was two (between 0 and 10), while in our study it was four (between 0 and 10). This suggests that while a similar proportion of adolescent report pain either often or all the time, those who identify as gender non-binary perceive greater intensity of pain. It was shown that adolescents who report persistent pain also have lower self-esteem, report higher level of perceived stress and loneliness, often complain about sleep problems, have more school absences, and overall experience a lower level of health-related quality of life as compared to adolescents with shorter pain durations (Mikkelsen et al., 2021). Increased pain intensity may worsen all these problem areas. In our study, 19% of the adolescents reported no experience of pain. This is in contrast with the Norwegian study where 24% of adolescents reported no pain. To summarize, a greater proportion of gender non-binary adolescents report pain of higher intensity than cisgender adolescents. The increased intensity of pain in our study group could be explained by the prevalence of physical disabilities in the sample (12%). This was double the figure reported in 2003 in the United States (5.8%) (Merrick and Carmeli, 2003). Physical disabilities are generally coupled with chronic pain (de la Vega et al., 2018). Therefore, we hypothesize that non-binary adolescents who are in pain have a lower health-related quality of life. In our study, pain intensity positively correlated with the level of psychological distress and negatively correlated with the level of alcohol use, which could suggest that alcohol may be used as self-medication to alleviate pain and psychological distress symptoms. This can be related to previous research concerning psychological distress and alcohol use among adolescents who identify beyond gender dichotomy (Livingston et al., 2016). Alcohol may be used as a strategy to cope with the negative aspects of life such as the stress of being a minority (Mereish, 2019). However, the correlation between pain, psychological distress, and alcohol is an interesting area for further research.

### 4.2. Psychological distress, mental health

Research shows that gender non-binary adolescents suffer from poor mental health and challenging living conditions to a much greater extent than the general population does (e.g.: Eisenberg et al., 2017; Chew et al., 2020; Paz-Otero et al., 2021; Surace et al., 2021). In the present study, a third of the study population (35%) reported the existence of at least one psychiatric diagnosis. This seems to be much higher than in cisgender males and females in a sample of Swedish adolescents between 15 and 19 years of age. Of the 1,594 respondents, 312 (19.6%) reported that they have at least one psychiatric diagnosis (unpublished data from the MeSHe project). The most common disorders in non-binary adolescents were depression and anxiety disorders (75% and 80% of cases, respectively vs. 46% and 51% found in the general Swedish population), followed by neurodevelopmental disorders (35% vs. 41% found in the general Swedish population), obsessive–compulsive behavior (20% vs. 3.7% found in the general Swedish population), post-traumatic stress disorder (PTSD; 10% vs. 6.8% found in the general Swedish population), self-harm and bipolar disorder (5% and 5% vs. 0.7% and 1% found in the general Swedish population). This data suggests that the prevalence of obsessive–compulsive disorder is remarkably increased in gender non-binary adolescents, and they also exhibit an increased prevalence of depression, anxiety disorders, self-harm, PTSD, and bipolar disorders. On the other hand, the prevalence of neurodevelopmental disorders (ADHD and ASD) are somewhat lower than a Swedish general population sample of adolescents of the same age (unpublished data). When assessing information on the type and level of psychological distress in our study population, the results were in accordance with the findings about psychiatric diagnoses. Such as, the most prominent distress factors in the non-binary adolescent’s life could be obsessive compulsive symptoms of BSI, depression, and interpersonal sensitivity. It is worth noting that BSI’s primary domains are not based on the DSM criteria. Therefore, for example, obsessive compulsive symptoms include problems such as “trouble remembering things, your mind going blank, trouble concentrating, difficulty in making decisions,” beside the more typical symptom of “having to check and double check what you do, difficulty in making decisions, your mind going blank.” Impaired decision making has not only been detected in participants with OCD, but it has been suggested that it can be a core feature of OCD (da Rocha et al., 2011). There is also evidence toward neuropsychological deficits in pediatric OCD populations considering visual memory and spatial working memory (Andrés et al., 2007; Bernardes et al., 2020). While some people with OCD complain about having difficulties concentrating, there is no demonstrated neurobiological overlap between OCD and ADHD. The overlap may be as simple as it being very difficult (if possible) to give full attention to more than one thing at the same time. While there are an increasing number of studies showing the co-existence of ADHD and OCD in pediatric samples, these studies explain their coexistence based on the deficit in impulse inhibition (see Brem et al., 2014).

BSI has been used over the past years to assess the psychological distress level in adolescents in the general population in Morocco (Zouini et al., 2019c) and Sweden (Kerekes et al., 2021a). In both the Moroccan (data collected 2013) and Swedish studies (data collected 2018), it has been shown that adolescents scored the highest on the obsessive–compulsive domain, which was followed by anxiety and interpersonal sensitivity or paranoid ideations. While the pattern was similar in both the countries, the level of psychological distress differed. Mzadi et al. (2022) showed that during the COVID-19 pandemic, the level of psychological distress significantly increased in the paranoid ideation and depression domains in Moroccan adolescents. As our data collection happened ~1 year into the pandemic, these results should indicate that gender non-binary adolescents report a similar pattern, but possibly different level of psychological distress, as gender binary adolescents. A recently published study from the MeSHe project including gender binary, 15–19-year-olds from the same multinational population, reports that during the pandemic, adolescents in different nations reported the highest psychological distress in similar patterns. Each nation’s sample scores demonstrated obsessive compulsive domains and interpersonal sensitivity within the three highest scores, followed by paranoid ideation and depression as nation-specific dominators (Sweden: depression, obsessive compulsive symptoms, interpersonal sensitivity; Morocco and Serbia: paranoid ideations, obsessive compulsive symptoms, interpersonal sensitivity; Vietnam: interpersonal sensitivity, obsessive compulsive symptoms, depression; and the United States: obsessive compulsive symptoms, depression and interpersonal sensitivity). Most importantly, while the pattern is similar, the level of distress differed between samples (American and Moroccan adolescents reported significantly higher psychological distress levels than those from Serbia, Sweden, and Vietnam; Nguyen et al., 2022). Our study population reported higher distress levels in obsessive compulsive symptoms, depression, and interpersonal sensitivity and in the General Severity Index than each of the other countries’ gender binary populations, except the United States. Generally, these results suggest that gender non-binary adolescents have similar stressors in their lives. They responded similarly to the COVID-19 pandemic as their gender binary counterparts, but with an increased level of distress.

### 4.3. Risk behaviors and aggression

In the present study, we assessed alcohol and drug use habits with two-two separate measures. Confirming the validity of each of the responses showed similar results. Over 60% of non-binary adolescents reported that they never used alcohol (on AUDIT and on COVID-related questions) and over 90% indicated that they have never used illegal drugs (on DUDIT and on COVID-related questions). These proportions are very similar to those found in a multinational sample of 15–19-year-old gender binary adolescents (Kerekes et al., 2021b). However, they differ from results obtained by Newcomb et al. (2020), who found high rates of risk behaviors such as substance use, in the population of *n* = 214 transgender and gender diverse adolescents in Chicago. The results of the present study showed fairly low levels of drug and alcohol use.

Importantly, in the present study, 12.1% indicated increased alcohol use and 5.2% indicated increased illegal drug use versus only 5.1% who indicated decreased alcohol consumption and 1.7% decreased their illegal drug use after the outbreak of the COVID-19 pandemic. Compared to the proportions in which these changes happened in gender binary adolescents, alcohol consumption: 10.2% increased and 13.3% decreased; illegal drug use: 3.2% increased and 2.1% decreased (Kerekes et al., 2021b), we can conclude that a greater proportion of non-binary adolescents who already used substances responded with increased use than decreased use. This was also higher compared to the proportion of gender binary adolescents who indicated increased substance use as a consequence of the pandemic. Risk behaviors such as using alcohol or drugs can be seen an example of coping with psychological distress. Hunt et al. (2021) found increased levels of psychological distress and lower psychological resilience among gender-diverse adolescents during the COVID-19 pandemic than their gender binary counterparts.

Beside substance use, we measured aggressive and antisocial behaviors demonstrated since the respondents turned 13 years old. The measure we used (AA-LHA), however, proved to not have cross-cultural validity, and researchers were warned not to use it for the comparison of aggressive and antisocial behaviors between genders (Stevanovic et al., 2022). Interestingly, the Hostility domain of the BSI was the only one that did not show validity in cross-cultural and cross-gender analyses (Nguyen et al., 2022). Therefore, we do not attempt to do any comparison with previous publications regarding non-binary adolescents’ aggressive and antisocial behaviors.

### 4.4. Victimization and negative psychosocial factors

Information about victimization was also assessed with independent measures in our study. Adolescents completed questions about both the frequency of different types of victimization in their life and how it has changed during the pandemic (as part of the COVID-related questions), but they also were asked in the first section of the MeSHe survey to respond if they ever experienced physical or psychological abuse. Half of the respondents indicated that they had experienced psychological abuse and 40% of indicated that they had experienced physical abuse at some time in their life. The experience of physical and psychological abuse had previously been shown to vary between genders, cultures, and the time of assessment (Kerekes et al., 2021a). While Kerekes et al. explained the high proportion of female adolescents (47%) reporting experiences of psychological abuse with the social environment in Sweden of the time (the “me too” movement in 2018), we can conclude that the proportion of non-binary adolescents reporting physical and/or psychological abuse is higher than reportage from any culture, at any time, with any gender adolescents (Zouini et al., 2019c; Kerekes et al., 2021a). Previous studies confirm that gender non-binary adolescents are much more vulnerable to victimization, discrimination, and bullying than the general population (Richards et al., 2016; Eisenberg et al., 2017; Rimes et al., 2019; Newcomb et al., 2020).

During the pandemic, 20% reported that they were physically abused compared to 10.3% in the multinational sample of gender binary adolescents, 25% reported that they were grouped or touched in a sexual manner without their consent compared to 12.2% of the gender binary people surveyed, and the same amount, 25%, were threatened and felt seriously afraid compared to 11.5% of the gender binary population (Kerekes et al., 2021b). In cyberbullying, 25% reported that someone had written offensive things about them online compared to 12.5% in the gender binary population and 16% compared to 8.9% in gender binary (Kerekes et al., 2021b) reported that someone had uploaded pictures or videos about them without their consent on the internet. This data shows an at least doubled risk of being victimized as a gender non-binary adolescent. This can be related to Rimes et al. (2019), who concluded that gender non-binary and binary transgender adolescents experienced higher levels of domestic violence and childhood sexual abuse than the gender binary population. The prevalence of health risk behaviors among transgender and gender non-conforming youths was described in Eisenberg et al. (2017), where in addition to suicidal ideations, they demonstrated greater risk of emotional stress and experiences of bullying with higher risk behaviors and lower protective factors compared to gender binary/cisgender youths.

While adolescence is a period of ontogenesis when individuals increase independency from their family/parents, the impact of the microenvironment (family) should be considered, especially during the COVID-19 pandemic when restrictions forced adolescents all over the world to stay home. In our study population, 17% reported living with adults with alcohol use problems, 40% also reported experiences of both physical and psychological abuse, while 40% reported experiencing neither physical nor psychological abuse. One adolescent reported living with an adult with a drug use problem and also reported experiencing of physical and psychological abuse. In previously published prevalence studies of parental alcohol and drug use problems, 0.4% of gender binary adolescents reported parental alcohol use problems in Sweden in 2018 (Kerekes et al., 2021a). 8.8% reported so in Morocco in 2013 (Zouini et al., 2019b,c). The results are thus dramatically higher in our study population. In terms of support from family and friends, the group of transgender and gender non-binary adolescents distinguished themselves from the cisgender population in terms of receiving less support from family and friends (Aparicio-García et al., 2018).

### 4.5. Affect states and personality profiles

Using the circumplex model of Watson and Clark (1994), we could detect that adolescents who identify as gender non-binary identity have a significantly higher level of negative than positive, mainly low-arousal, affect states. They indicated the dominance of feelings of sadness, guilt, and shame in their lives, which is in concordance with Aparicio-García’s et al. (2018) study, where non-binary adolescents rated higher levels of being unhappy, feeling isolated, and having thoughts of suicide than adolescents who identify as transgender or cisgender. If high positive affects combined with low negative affects result in greater subjective well-being experiences (Jayawickreme et al., 2012), than we can conclude that our study population, with high negative affect in combination with low positive ones, may indicate unwellness in non-binary adolescents. The high level of negative affects reported in our study mirrored the high levels of self-rated depression and interpersonal sensitivity. The dominance of negative emotions in the adolescents’ lives also mirrored their personality profiles. We found that non-binary adolescents can be described with the representative personality traits of Openness and Neuroticism, which are closely followed by Agreeableness. The previously presented results, high scores of negative emotions, dominance of obsessive compulsiveness, depression, and interpersonal sensitivity symptoms, often present diagnoses of anxiety, depression, OCD, PTSD, and self-harm disorders, high levels of victimization and high frequencies of familiar negative psychosocial factors. The high score on Neuroticism is thus the easiest to understand. People with high Neuroticism have high scores of anxiety, depression, anger, self-consciousness, and emotional lability (Duggan et al., 1990; Boyce et al., 1991). In the comprehensive study by Weisberg et al. (2011), it is pointed out that gender differences are difficult to notice in the primary domains of the Big Five, however they exist in Neuroticism, Agreeableness, and Extroversion (but not Openness) when comparing male and female genders. Gender differences decrease with increasing age for Neuroticism but increase for Agreeableness (Weisberg et al., 2011). This may suggest that our study population has a unique composition of personalities dominated by intellectual curiosity, creative imagination, and an appreciation of aesthetic experiences, in combination with anxious depressive personality traits and emotional lability, flavored with altruism, empathy, and kindness. This personality profile can be a productive and important part of society, and when protective instances are applied for everyday safety, can serve to increase the existing level of understanding and respect for adolescents who do not identify with the male or female genders.

## 5. Future challenges: Challenges for academic studies and challenges for care professionals, social workers, educators, and families

Multidisciplinary mental health professionals and teams are needed, as well as clinical guidelines and treatment protocols, to meet the complexity of non-binary people’s situations and needs (Richards et al., 2016). The results and discussion of this study indicate the level, but not the pattern of psychiatric distress, and the prevalence, but not the type of psychiatric disorders that differ in non-binary and gender binary adolescents. A general adaptation, special development of care, and deeper and broader knowledge of non-binary adolescents are thus needed. More studies are required with an increased focus on a more diversified picture in terms of gender identification in collaboration with local and national organizations. Furthermore, the development of appropriate support measures in healthcare, education systems, authorities, as well as voluntary organizations are required. The results strengthen the increased demands that exist for faster adaptation and development of care and social support in accordance with non-binary individuals’ experiences and needs (Moore et al., 2021). This also applies to their micro- and meso-environments, where the support of families and friends is important for adolescents’ psychological health and resilience (Weinhardt et al., 2019).

In academic methodologies, the potential consequences of offering only two genders to choose from, when gender identity is requested in studies, should be considered. Even if the purpose of the study determines the design of alternative gender definitions, self-identification can be recommended. The existence of a gender category to identify with is essential for many people, at least if categorization processes are fundamental to the social organization and understanding of both individuals and society at large. Research processes establish social categorization, so not letting a respondent self-identify their gender signals that a growing number of people will be defined as non-existent and incomprehensible, which will be perceived as offensive (Butler, 1990, 1993). It also means that knowledge about gender variations will remain invisible. Furthermore, it might serve to stabilize, normalize, and reproduce the bi-gendered norms that still dominate society along with its negative effects.

## 6. Strengths and limitations

One of the greatest strengths of the present study is its study population. We focused solely on the group that actively defined themselves as neither male nor neither female gender (not those who did not respond to the question about gender) in a multinational sample of 15–19-year-old adolescents. The prevalence of adolescents identifying with genders other than strictly male, or female is increasingly recognized in today’s societies (Richards et al., 2016). Since this group differs on several points from the other two gender groups, it is of essential importance to increase our knowledge about their well-being, vulnerability, and strength and potential in society. Kerekes et al. (2021b). However, the strength of the study population also contains a weakness, the weakness is the definition of the concept of non-binary gender. In this study, we have categorized those, who selected “neither of these” as their gender from the possible options (female/male/neither of these), as non-binary. Transgender or gender fluid categories could not be identified as specific categories based on the information we have collected, which is a limitation in the design of the study.

While the low number of participants could be considered as a limitation of the study, which is reflected in the high confidence intervals of the population proportions, it mirrors the actual prevalence of gender non-binary people from a multinational, general population sample of almost 6,000 adolescents. Therefore, the use of a multinational and general population sample is also a strength of the present study. In addition, the low prevalence of non-binary participants shows the importance of taking groups consisting of a few people seriously, since otherwise they risk being neglected by statistics and consequently excluded in research. The use of self-reported measures could be considered both a strength and a limitation. An undoubted limitation of the study is its cross-sectional design, which prevents analyses of causality between emerging aspects.

## Data availability statement

The raw data supporting the conclusions of this article will be made available upon request without undue reservation, by the authors.

## Ethics statement

The studies involving human participants were reviewed and approved by each participating country’s relevant national or institutional ethical board, which are: Sweden: National Review Board (Drn: 689-17 and 2020-03351); Serbia: The Ethics Committee of the Clinic for Neurology and Psychiatry for Children and Youth Belgrade; Morocco: Regional Directorate of the Ministry of National Education in Tetouan (authorization number 85); Vietnam: Review Board of Centre for Assisting and Consulting Psychology, University of Social Sciences and Humanities, Hanoi (RPSY-101); USA: Institutional Review Board for the Protection of Human Subjects, SUNY Upstate Medical University (1651637-32020-E). Written informed consent from the participants’ legal guardian/next of kin was not required to participate in this study in accordance with the national legislation and the institutional requirements.

## Author contributions

The project was planned and led by NK, who is the principal investigator. KB has built the electronic survey and synchronized it for each language. KB and NK made the statistical analyses. CJ was responsible for the drafting of the manuscript. CJ, CK, and NK were responsible for developing the manuscript. Each co-author participated in finalizing the manuscript. All authors contributed to the article and approved the submitted version.

## Conflict of interest

The authors declare that the research was conducted in the absence of any commercial or financial relationships that could be construed as a potential conflict of interest.

## Publisher’s note

All claims expressed in this article are solely those of the authors and do not necessarily represent those of their affiliated organizations, or those of the publisher, the editors and the reviewers. Any product that may be evaluated in this article, or claim that may be made by its manufacturer, is not guaranteed or endorsed by the publisher.

## Footnotes

^1^ www.meshe.se
